# Supplementary material for: CytoCellDB: a comprehensive resource for exploring extrachromosomal DNA in cancer cell lines
Source: NAR Cancer. 2024 Aug 1;6(3):zcae035. doi: 10.1093/narcan/zcae035 (PMC11292414; doi:10.1093/narcan/zcae035)
Supplement: zcae035_Supplemental_Files [file zcae035_supplemental_files.zip › CytoCellDB_Supplementary (1).pdf]

CytoCellDB: A Comprehensive Resource for Exploring Extrachromosomal DNA in Cancer Cell Lines

Jacob Fessler[*,a], Stephanie Ting[*,c], Hong Yi[h], Santiago Haase[d], Jingting Chen[b], Saygin Gulec[g], Yue Wang[e], Nathan Smyers[g], Kohen Goble[c], Danielle Cannon[c], Aarav Mehta[a], Christina Ford[d], Elizabeth Brunk[*,c,d,e,f,i,†]

[a] Department of Computer Science, University of North Carolina at Chapel Hill, Chapel Hill, NC 27516

[b] Department of Biochemistry and Biophysics, University of North Carolina at Chapel Hill, Chapel Hill, NC 27516

[c] Department of Chemistry, University of North Carolina at Chapel Hill, Chapel Hill, NC 27516

[d] Integrative Program for Biological and Genome Sciences (IBGS), University of North Carolina at Chapel Hill, Chapel Hill, NC 27516

[e] Department of Pharmacology, University of North Carolina at Chapel Hill, Chapel Hill, NC 27516

[f] Computational Medicine Program, University of North Carolina at Chapel Hill, Chapel Hill, NC 27516

[g] Curriculum in Bioinformatics and Computational Biology, University of North Carolina at Chapel Hill, Chapel Hill, NC 27516

[h] Renaissance Computing Institute (RENCI), University of North Carolina at Chapel Hill, Chapel Hill, NC 27516

[i] Lineberger Comprehensive Cancer Center, University of North Carolina at Chapel Hill, Chapel Hill, NC 27516

† Correspondence should be addressed to: Elizabeth Brunk (elizabeth_brunk@med.unc.edu); *These authors contributed equally

Keywords

Cytogenetics, DepMap, CCLE, Extrachromosomal DNA, ecDNA, double minute chromosomes, machine learning, multi-omics, resource

Supporting Information

Supp File 1| Excel Spreadsheet containing the following information:

Sheet 1: Master Data Frame of CytoCellDB

Sheet 2: 237 engineered karyotype features from CytoCellDB

Sheet 3: 237 engineered karyotype features from MitelMan Database

Sheet 4: RNA and CNV percentiles for DepMap Cell lines

Jupyter Lab Notebooks | ipython notebooks as supplementary material include:

1. Karyotype extraction from DepMap and MitelMan Databases
2. Machine Learning Models using karyotype data
3. Data analytics of pairwise CNV and RNA relationships

Sup Fig. 1| Representative pathways that differentiate ecDNA+ and ecDNA- cells.

Sup Fig. 2| SHAP Plots for Feature Directionality

Sup Fig. 3| Functional Clustering Approach with examples

Supp Table 1| Pairwise CNV and RNA plots identify key genes potentially amplified on ecDNA

Supp Table 2| Pairwise CNV and RNA plots identify key genes potentially amplified on HSR

Supp Table 3| HSR+ Relationship between karyotype confidence scores, CNV and ecDNA/HSR classification.

Supp Table 4| Relationship between CNV and ecDNA/HSR classification

Detailed Methods

- **Running Amplicon Architect**
- **Running CircleHunter**

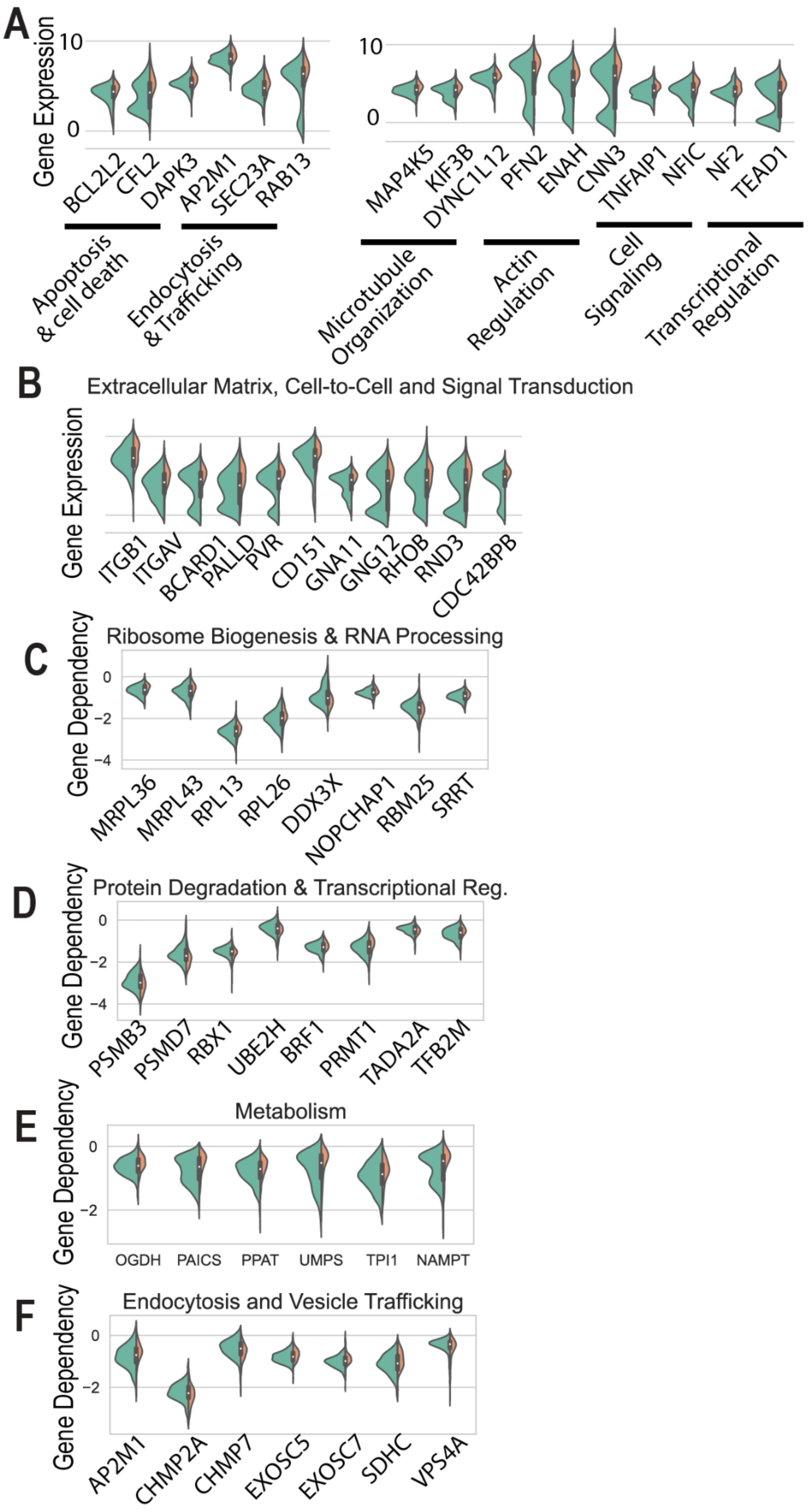
A
Gene Expression
10
0
BCL2L2
CFL2
DAPK3
AP2M1
SEC23A
RAB13
Apoptosis & cell death
Endocytosis & Trafficking
10
0
MAP4K5
KIF3B
DYNC1L12
PFN2
ENAH
CNN3
TNFAIP1
NFIC
NF2
TEAD1
Microtubule Organization
Actin Regulation
Cell Signaling
Transcriptional Regulation
B
Extracellular Matrix, Cell-to-Cell and Signal Transduction
Gene Expression
ITGB1
ITGAV
BCARD1
PALLD
PVR
CD151
GNA11
GNG12
RHOB
RND3
CDC42BPB
C
Ribosome Biogenesis & RNA Processing
Gene Dependency
0
-2
-4
MRPL36
MRPL43
RPL13
RPL26
DDX3X
NOPCHAP1
RBM25
SRRT
D
Protein Degradation & Transcriptional Reg.
Gene Dependency
0
-2
-4
PSMB3
PSMD7
RBX1
UBE2H
BRF1
PRMT1
TADA2A
TFB2M
E
Metabolism
Gene Dependency
0
-2
OGDH
PAICS
PPAT
UMPS
TPI1
NAMPT
F
Endocytosis and Vesicle Trafficking
Gene Dependency
0
-2
AP2M1
CHMP2A
CHMP7
EXOSC5
EXOSC7
SDHC
VPS4A

Supp Fig 1. Comparing differentially expressed genes and genes with significantly different gene dependency scores in ecDNA+ versus ecDNA- cell lines. Gene expression differences from various molecular pathways including **A.** Apoptosis, endocytosis, trafficking, microtubule, actin, cell signaling, and transcriptional regulation. **B.** extracellular matrix, cell to cell signaling, and signal transduction. **C.** Gene dependency differences from various molecular pathways including **C.** ribosome biogenesis and RNA processing, **D.** protein degradation and transcriptional regulation, **E.** Metabolism and **F.** endocytosis and vesicle trafficking.

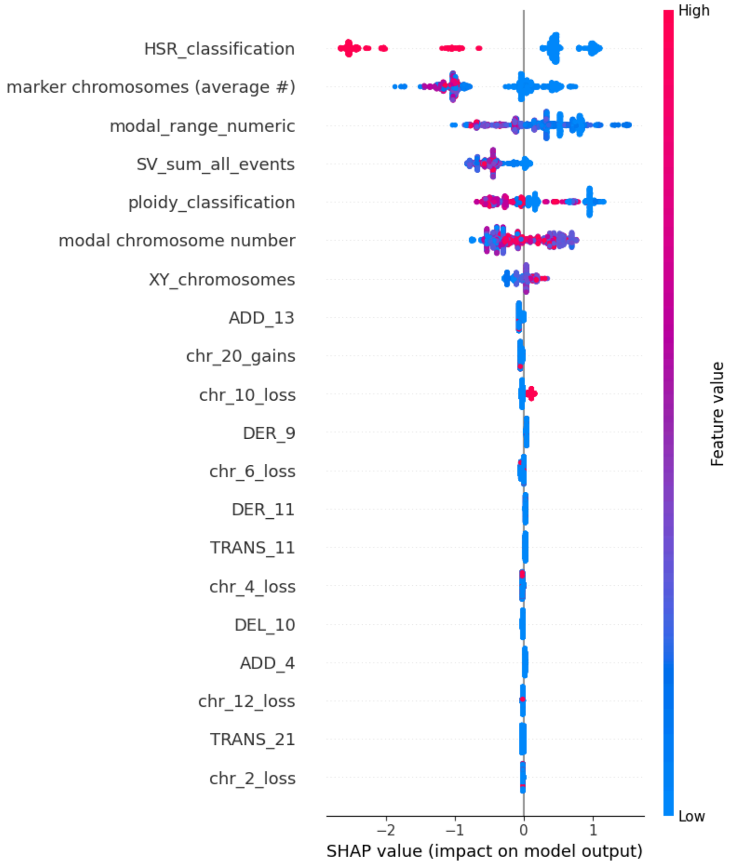

Supp Fig 2. The SHAP plot. The features listed at the top are the most important. The wider the spread of SHAP values for a feature, the more varied its impact on the model's predictions. A feature with both negative and positive SHAP values means it can both increase and decrease the predicted value depending on the instance. If high values of a feature (red points) are associated with positive SHAP values (right side), it means higher values of that feature increase the model's prediction. Conversely, if red points are on the left, higher values of the feature decrease the prediction. By examining the SHAP summary plot, we can understand not only which features are important but also how they influence the model's predictions in different ways.

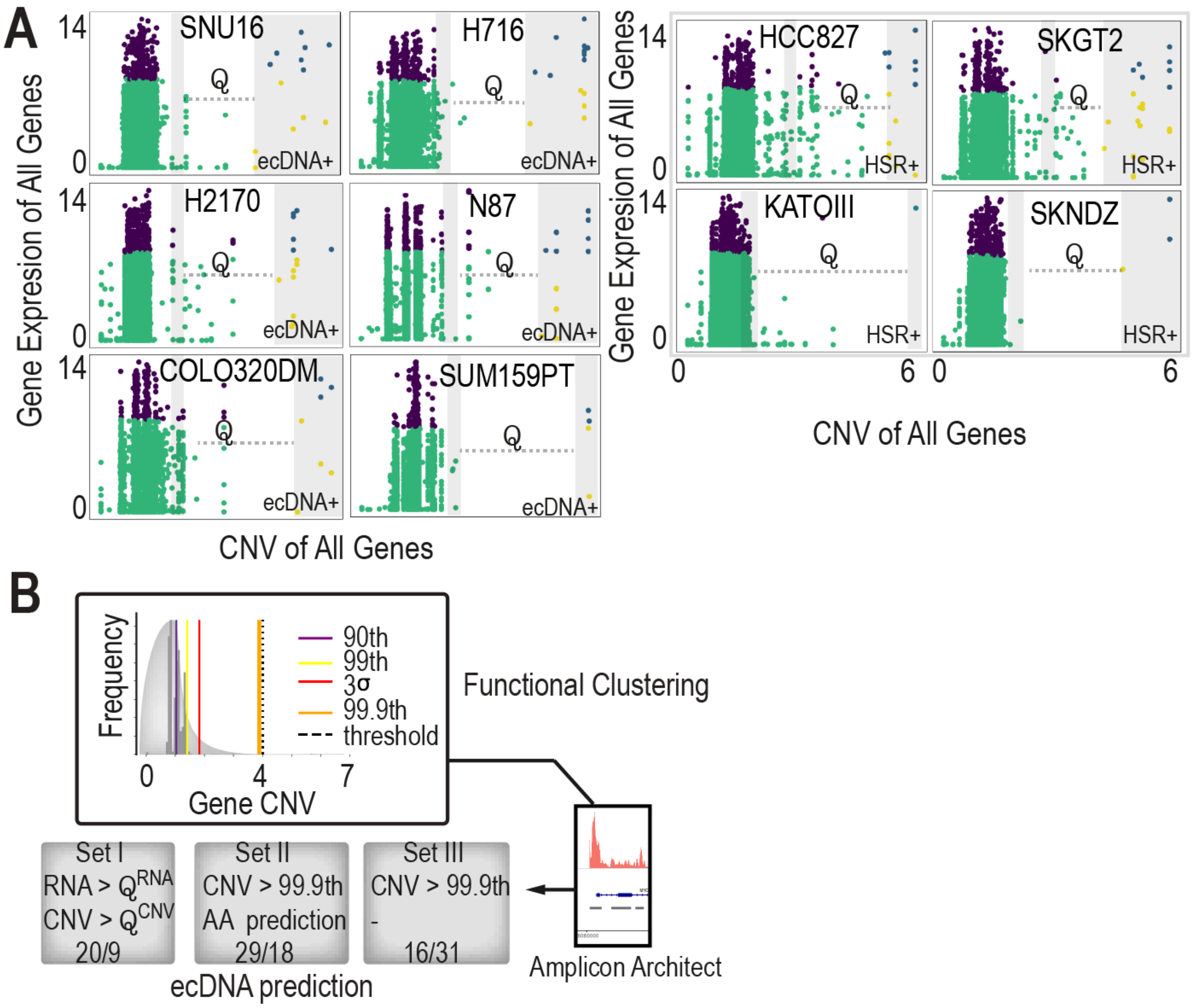

Supp Fig 3. A. A parameter Q was derived to functionally differentiate genes amplified and expressed from ecDNA versus from HSR. Plotted are pairwise comparisons between gene expression (all genes within a cell line sample) versus copy number (of all genes within a cell line sample). Labeled are cell lines annotated as ecDNA+ or HSR+. **B.** Functional clustering approach: using a percentile-thresholding based approach, clusters of RNA vs CNV are defined, the parameter Q is computed and cell lines are selected as candidates for ecDNA expression. Additionally, predictions from AmpliconArchitect are integrated to create a multi-tiered, multi-omics approach.

Supp Table 1. EcDNA+ Cell lines with Functional Clustering Patterns

Index	CCLE_Name
0	NCIH889_LUNG
7	NCIH1694_LUNG
15	NCIH1963_LUNG
25	NCIH510_LUNG
34	HCC33_LUNG
44	NCIH1092_LUNG
102	SNU16_STOMACH
109	NCIH716_LARGE_INTESTINE
143	SNUC1_LARGE_INTESTINE
196	RH18_SOFT_TISSUE
344	DKMG_CENTRAL_NERVOUS_SYSTEM
346	CCFSTTG1_CENTRAL_NERVOUS_SYSTEM
352	COLO320_LARGE_INTESTINE
357	SUMB002_CENTRAL_NERVOUS_SYSTEM
376	NCIN87_STOMACH
429	NCIH2170_LUNG
439	HCC1569_BREAST
454	Y79_AUTONOMIC_GANGLIA
458	CHP212_AUTON

	OMIC_GANGLIA
**467**	NCIH526_LUNG
**474**	SIMA_AUTONOMIC_GANGLIA
**476**	CHP126_AUTONOMIC_GANGLIA
**488**	NCIH69_LUNG
**490**	LAN2_AUTONOMIC_GANGLIA
**517**	NCIH1395_LUNG
**536**	SUM159PT_BREAST
**538**	CORL311_LUNG
**571**	8305C_THYROID
**572**	NCIH2171_LUNG
**585**	NCIH446_LUNG
**586**	D341MED_CENTRAL_NERVOUS_SYSTEM
**587**	MSTO211H_PLEURA
**588**	NCIH82_LUNG
**591**	SCLC21H_LUNG
**592**	DMS273_LUNG
**593**	NCIH524_LUNG
**595**	SKPNDW_BONE
**596**	SCLC22H_LUNG

Supp Table 2. HSR+ Cell lines with Functional Clustering Patterns

**CCLE_Name**	
**15**	NCIH1963_LUNG
**25**	NCIH510_LUNG

**102**	SNU16_STOMACH
**120**	KATOIII_STOMACH
**126**	MFM223_BREAST
**230**	SJSA1_BONE
**252**	HCC827_LUNG
**346**	CCFSTTG1_CENTRAL_NERVOUS_SYSTEM
**406**	CALU3_LUNG
**450**	HUPT3_PANCREAS
**462**	IMR32_AUTONOMIC_GANGLIA
**481**	SKNDZ_AUTONOMIC_GANGLIA
**485**	MHHNB11_AUTONOMIC_GANGLIA
**487**	KELLY_AUTONOMIC_GANGLIA
**515**	MCF7_BREAST
**521**	OV90_OVARY
**566**	ESO51_OESOPHAGUS
**585**	NCIH446_LUNG
**586**	D341MED_CENTRAL_NERVOUS_SYSTEM
**588**	NCIH82_LUNG

Supp Table 3. HSR+ Relationship between karyotype confidence scores, CNV and ecDNA/HSR classification.

	Total # cell lines	Total Positive ecDNA / HSR	Total Negative ecDNA / HSR	Total Unclassified

0 - No data	1			1
1 - Low Confidence	53			53
2 - Medium Conf.	204		204	
3 - High Conf	192	**142**	17	33

Cell lines lacking experimentally validated ecDNA or HSRs but having amplified oncogenes likely harbor ecDNA or HSRs that have yet to be confirmed. Many cell lines may fit this description, or have predicted ecDNA/HSRs from algorithms like AmpliconArchitect or CircleHunter. CytoCellDB reports validated ecDNA and/or HSR presence from public cytogenetic data, but this data often does not explicitly confirm ecDNA. We classify a cell line as ecDNA-positive if the karyotype includes terms like “double minutes,” “dmins,” “minute chromosomes,” etc. Some karyotype data may not provide enough detail for confident classification, and we have labeled the confidence of the karyotype data based on the details provided.

Another possibility is that smaller amplifications are not cytogenetically significant. For example, cell lines with only 2-3 ecDNA in a low percentage of cells may have lower-level amplifications, making them harder to detect. Depending on the thoroughness of the cytogenetic analysis, ecDNA may be missed in cases with few ecDNA per cell and a lower percentage of cells harboring ecDNA.

To address this point, we provide additional data showing the relationships between karyotype confidence, amplification levels, and ecDNA/HSR classifications. Supp Table 3 displays karyotype confidence scores (0-3) and their distribution across ecDNA/HSR-positive, ecDNA/HSR-negative, and unclassified cell lines. All cell lines in this analysis had amplifications greater than a log2 CNV value of 2.

In the highest confidence category, most cases are classified as ecDNA/HSR positive. This category includes data with direct experimental evidence (images) or explicit karyotype annotations indicating ecDNA or HSR presence. The medium confidence category has the highest number of ecDNA/HSR-negative cases but should be interpreted with caution. Medium confidence is assigned when karyotype data do not explicitly detail ecDNA/HSRs but provide specific information on other features (e.g., chromosome losses, gains, and marker chromosomes). Thus, we classify these cases as “negative” but with only medium confidence.

Supp Table 4 indicates that the distribution of CNV value also impacts the percentage of cell lines classified as ecDNA/HSR positive versus negative. For this analysis, we considered a cell line’s max CNV of all protein coding genes. As expected, higher copy numbers provide better evidence in FISH images, as more cells within the population likely present ecDNA or HSRs. In contrast, CNV between 2-3 indicates lower-level amplifications that might be more difficult to detect in karyotype

FISH images. Follow-up experiments on cell lines with high amplifications and negative ecDNA/HSR classifications would be interesting and important.

Supp Table 4. Relationship between CNV and ecDNA/HSR classification.

CNV threshold	Total Positive ecDNA / HSR	Total Negative ecDNA / HSR	Total cell lines within CNV range
2-3	43	122	200
3-4	39	81	154
4-5	33	17	64
5-6	17	2	22
6-7	10	0	11

Detailed Methods

Running Amplicon Architect

The AmpliconArchitect pipeline begins with sequencing parameter estimation to understand the overall quality and distribution of sequencing data, followed by CNV boundary detection to delineate regions of the genome with varying copy numbers. After this step, amplified intervals are filtered based on a predefined threshold of copy number gain. To define amplified intervals, a “Gain” parameter is set, which determines the relative coverage of the region in consideration with respect to the genome median coverage. We did not modify the default parameters of the function, which are Gain = 4.5 and CNSIZE_MIN = 50,000 (minimum size of the amplified region in consideration), as specified in the AmpliconSuite GitHub webpage (https://github.com/AmpliconSuite/AmpliconSuite-pipeline/blob/master/documentation/GUIDE.md):

"We recommend picking regions which have an estimated CN >= 4.5 and size > 50 kbp, which do not appear amplified due to being parts of repeat elements, and which are not amplified due to karyotypic abnormality."

After this, AmpliconArchitect detects the CNV boundaries and the initial seed intervals. AA identifies amplified regions by iteratively extending the intervals based on coverage thresholds. After setting the “Gain” parameter, AmpliconArchitect applies a dynamic threshold based on the formula “(GAIN + 2 * max(1.0, chrom_cov_ratio) - 2)” where “GAIN” is the base gain threshold set by the user (default = 4.5) and “chrom_cov_ratio” is the ratio of the median coverage of the interval to the median coverage of the whole genome. This formula adjusts the threshold based on the coverage ratio. If the interval has higher coverage relative to the genome-wide coverage, the threshold increases, making the filter more stringent.

After filtering and selecting the initial set of amplified intervals based on the dynamic threshold, AmpliconArchitect (AA) iterates to identify and add all connected amplified fragments. AA starts with the seed intervals that have been identified based on the initial filtering criteria, and then uses an iterative approach to search for all connected amplified intervals starting from the seed intervals. The process involves creating a max-heap data structure to manage intervals during the search. In each iteration, AA selects an interval from the heap and identifies discordant read pair biclusters associated with it. AA then attempts to extend these biclusters by querying whether the extended portion is amplified.

The script evaluates whether a fragment should be added to an amplified interval by calculating the median coverage of the fragment and comparing it to the existing intervals and the genome. It dynamically adjusts the gain threshold based on the median coverage ratios to ensure that only fragments with significant coverage increases are added. This approach helps in refining and accurately identifying amplified intervals based on their coverage profiles.

After the intervals are filtered and merged, the next step involves identifying breakpoints within these intervals. Discordant reads are identified and clustered based on their mapping positions. Clusters are filtered to retain significant breakpoints. The detected breakpoints are used to construct a breakpoint graph, representing the structural variations within the amplified regions. Finally, the breakpoint graph is decomposed into simple cycles to elucidate the structure of the amplicons.

Overall, to define an ecDNA, AmpliconArchitect does not rely solely on coverage. It begins with building a map of amplified regions that have increased coverage compared to the genome and generates amplified intervals within the same chromosome based on coverage thresholds and other criteria. The process iteratively compares the added fragments to the amplified region being constructed. After this step, the information on the discordant reads (and the coverage of these reads) is used to merge amplified intervals into amplicons (which might encompass different chromosomes or circular structures). The criteria to identify ecDNA and eccDNA are based on the fulfillment of all these conditions.

Running CircleHunter

We run Circlehunter without any modifications from the default settings.

Circlehunter does not rely on coverage thresholds, but as it was conceived to predict ecDNA from bulk ATAC-seq it uses peak calling to identify enriched (amplified) regions instead.

All reads are used as input for peak calling using the tool MACS2. This tool identifies regions of the genome where the number of reads mapping to that region (read depth) is significantly higher than expected by chance. For each base in the genome, MACS2 calculates the P value, which represents the probability that the observed read depth at that base could occur by random chance. Bases with a P value less than 0.05 are considered significant. This threshold

means that there is less than a 5% chance that the observed read depth at these bases is due to random variation alone.

Peaks that are less than 12.5 kb apart are merged into a single consecutive enrich region using Bedtools, ensuring that nearby significant peaks are treated as a unified region, reflecting the biological significance of clusters of enriched reads.

As CircleHunter calculates directly the P value of enrichment comparing the base coverage with the background, it does not rely on a coverage threshold.

After calculating the enriched regions, CH extracts discordant read pairs, defined as pairs where the insert size is greater than 1500 bp (default, but adjustable), and are extracted from the mapped reads. These discordant pairs suggest structural variations or large genomic rearrangements. These extracted discordant read pairs are then piled up, and the ratio of their depth to the total read depth at each base is calculated. This ratio reflects the relative enrichment of discordant pairs at specific bases compared to the overall genome. Only discordant enrich regions that overlap with previously identified consecutive enrich regions are retained.

After this, the filtered discordant reads are used to construct a breakpoint graph where all discordant read pair enrich regions are added as nodes. Two types of edges are then introduced based on the nature of the enriched regions. Discordant type edges connect regions that share paired end reads with the same read ID, and a minimum number of these reads must be present to form such an edge. The default setting for this minimum is the inverse survival of the Poisson model used for enrichment assessment. When a discordant type of edge is added, the orientation of the supporting reads is recorded, which is used to determine the extension direction of the node. Consecutive type edges connect nodes that are properly oriented within one enrich region, reflecting contiguous genomic regions.

To estimate the precise breakpoint positions, Circlehunter employs a Bayesian model that integrates two distinct Bayesian approaches. The first model assumes that discordant read pairs mapped in the discordant enrich region can only estimate the minimum extended position of the breakpoint. This model uses a uniform distribution. The second model assumes that reads aligned to breakpoints will be clipped during alignment, with their end positions falling within a small distance from the actual breakpoint, modeled as a normal distribution. The combined posterior probabilities from these models provide a robust estimate of the breakpoint position. The maximum-likelihood estimates and 95% confidence intervals are obtained through a grid search, and the estimated breakpoints are applied to the graph nodes.
Finally, the search for ecDNA structures from the breakpoint graph is conducted using a modified depth-first search algorithm. This algorithm navigates the graph by alternating between the two types of edges, ensuring that the path connecting breakpoints includes both discordant and consecutive types. The search prioritizes segments with larger sizes and higher local depth to include more genes and find more reliable ecDNA structures. The results, including all possible circles and breakpoint confidence intervals, are output in BED format. For complex

rearrangements, the output can be restricted to a default of 1000 samples to manage the number of alternative sub-structures.

In summary, Circlehunter is a comprehensive pipeline that combines the identification of enriched regions, constructs a breakpoint graph, and estimates precise breakpoints using advanced statistical models.
